# CD157 in bone marrow mesenchymal stem cells mediates mitochondrial production and transfer to improve neuronal apoptosis and functional recovery after spinal cord injury

**DOI:** 10.1186/s13287-021-02305-w

**Published:** 2021-05-17

**Authors:** Jing Li, Heyangzi Li, Simin Cai, Shi Bai, Huabo Cai, Xiaoming Zhang

**Affiliations:** 1 Department of Anatomy, School of Medicine, Zhejiang University, Hangzhou, 310058 China; 2 Department of Anatomy, Taizhou University; School of Medicine, Zhejiang University, 310058 Hangzhou, China; 3 Department of Emergency Medicine, Sir Run Run Shaw Hospital; School of Medicine, Zhejiang University, Hangzhou, 310058 China

**Keywords:** Spinal cord injury, Bone marrow stromal cells, Mitochondria, CD157, Calcium

## Abstract

**Background:**

Recent studies demonstrated that autologous mitochondria derived from bone marrow mesenchymal stem cells (BMSCs) might be valuable in the treatment of spinal cord injury (SCI). However, the mechanisms of mitochondrial transfer from BMSCs to injured neurons are not fully understood.

**Methods:**

We modified BMSCs by CD157, a cell surface molecule as a potential regulator mitochondria transfer, then transplanted to SCI rats and co-cultured with OGD injured VSC4.1 motor neuron. We detected extracellular mitochondrial particles derived from BMSCs by transmission electron microscope and measured the CD157/cyclic ADP-ribose signaling pathway-related protein expression by immunohistochemistry and Western blotting assay. The CD157 ADPR-cyclase activity and Fluo-4 AM was used to detect the Ca^2+^ signal. All data were expressed as mean ± SEM. Statistical analysis was analyzed by GraphPad Prism 6 software. Unpaired *t*-test was used for the analysis of two groups. Multiple comparisons were evaluated by one-way ANOVA or two-way ANOVA.

**Results:**

CD157 on BMSCs was upregulated when co-cultured with injured VSC4.1 motor neurons. Upregulation of CD157 on BMSCs could raise the transfer extracellular mitochondria particles to VSC4.1 motor neurons, gradually regenerate the axon of VSC4.1 motor neuron and reduce the cell apoptosis. Transplantation of CD157-modified BMSCs at the injured sites could significantly improve the functional recovery, axon regeneration, and neuron apoptosis in SCI rats. The level of Ca^2+^ in CD157-modified BMSCs dramatically increased when objected to high concentration cADPR, ATP content, and MMP of BMSCs also increased.

**Conclusion:**

The present results suggested that CD157 can regulate the production and transfer of BMSC-derived extracellular mitochondrial particles, enriching the mechanism of the extracellular mitochondrial transfer in BMSCs transplantation and providing a novel strategy to improve the stem cell treatment on SCI.

## Introduction

Spinal cord injury (SCI) is a devastating disease with complex secondary pathological complications and it still has no effective treatment. SCI causes a series of complex pathological changes, including ischemia [1, 2], oxidative stress [3], neuroinflammation [4], axonal demyelination and degeneration [5], apoptosis [6], and spinal cord injury-induced immune deficiency syndrome after initial trauma [7, 8]. Furthermore, spinal cord injury-induced immune deficiency syndrome, excessive inflammatory responses [9], ionic homeostasis loss [10, 11], the upregulated release of excitatory amino acids, and the excess excitatory amino acid receptor activation can cause secondary injury cascades after SCI [12]. The study aimed to determine the neuron recovery and axon regeneration for spinal cord injury treatment.

The mitochondrion is one of the most important and studied subcellular organelles and its morphology and function are greatly influenced by secondary injury after SCI [13]. Mitochondria function and morphology changes, including irregular shape, enlarged size, disordered cristae, fusion and fission, reduced membrane potential, and expression changes of related proteins occur in the acute phase after SCI. Furthermore, these morphological and functional changes regulate underlying secondary injury processes, such as necrosis, apoptosis, and autophagy [14]. Mitochondrial dysfunction influences secondary injury development and neuronal cell death [13, 15]. Therefore, restoring the mitochondria function could be a potential therapeutic SCI strategy.

Bone marrow mesenchymal stem cells (BMSCs) from bone marrow with excellent capacity to differentiate into multiple cell lineages have served as an ideal tool for our SCI studies [16]. Of late, BMSCs have been frequently used for transplantation studies because they are easy to harvest, culture, expand, and modulate in vitro [17, 18]. BMSCs have various characteristics hence their versatility in the injured tissue’ repair mechanisms [19]. For example, BMSCs can improve bone regeneration to repair lost bone [20] and differentiate into cardiomyocytes [21]. Our previous studies found that BMSCs transplantation after SCI improved locomotor function in SCI rats, alleviated pathological deterioration through mitochondria transfer, and decreased ER stress-induced neuronal apoptosis and its related factors [22].

CD157 (bst-1, bone marrow stromal antigen-1) is a cell surface molecule expressed in myeloid, endothelial, mesothelial, and epithelial ovarian cancer cells, and acts as an ectoenzyme and a signaling receptor [23]. CD157 is also capable of signal transduction [24]. Reports have shown that CD157 promotes pre-T cell expansion [25] and regulates leukocyte trafficking [26] and ovarian cancer progression [22]. It is also involved in humoral immune responses [27], neutrophil transmigration [28], and hematopoietic stem cell support [29]. Besides, CD157 (a member of the NADase/ADP-ribosyl cyclase family) has been reported to induce cyclic ADP-ribose catalysis in embryonic and adult nervous systems [30]. This study hypothesized that CD157 regulates mitochondria transfer from BMSCs to injured neurons after SCI and promotes motor function recovery and axon regeneration via the CD157/cADPR/calcium signaling pathway.

## Materials and methods

### Animals

Male Sprague-Dawley rats sacrificed for this study. All experimental procedures were approved by the Animal Ethics Committee of Zhejiang University and followed the National Institutes of Health guidelines strictly. Animals were housed under a 12-h light/dark cycle with free access to food and water. All efforts were made to minimize the number of animals used and their suffering. The individual mouse was considered the experimental unit within the studies.

### Primary BMSCs isolation, culture, and characterization

Primary BMSCs were isolated from the femurs of 3–4 week old Sprague-Dawley male rats following our previous study. BMSCs were cultured in Dulbecco’s modified Eagle medium: Nutrient Mixture F-12 (DMEM/F-12) with 10% fetal bovine serum, 100 U/ml penicillin and 100 U/ml streptomycin, and medium were changed every 2 days. The fluorescence-activated cell sorting (FACS) analysis was adopted to characterize BMSCs in our previous study [31].

### Construction of adenovirus and infection of BMSCs

The CD157 overexpression vector was constructed with pHBAD-EF1-MCS-3flag-CMV-GFP vector. The MCS segment was inserted bst-1 gene, and the EF1 promoter regulated the expression of bst-1 gene and the CMV promoter regulated EGFP. Meanwhile, pHBAd-U6-MCS-CMV-GFP was used to construct CD157 interference vector that contained a U6 promoter which regulated the expression of shRNA of bst-1 inserted in the EcoR I and BamH I sites and a CMV promoter regulated the expression of GFP gene. A vector only expressing GFP gene was used to be the control. All vectors were synthesized by the Han Bio Co. LTD (Shanghai, China).

The BMSCs were infected at a 50 multiplicity of infection with the adenovirus vectors after the polybrene (5 μg/ml) treatment for 30 min. Forty-eight hours later, the infected BMSCs were observed with fluorescence microscope (Olympus Corp., Tokyo, Japan) and the Western blotting analysis was applied to detect the expression of CD157.

### Oxygen-glucose deprivation (OGD) and re-oxygenation of VSC4.1 motor neurons

The ventral spinal cord 4.1 (VSC4.1) motor neuron cells were cultured in RPMI 1640 medium with 10% (V/V) fetal bovine serum and 100 U/m penicillin and streptomycin at 37 °C with 5% CO_2_ in a fully humidified incubator. OGD and re-oxygenation models were used to mimic ischemia and hypoxia in SCI. In brief, VSC4.1 motor neurons were cultured in D-Hanks’ balanced salt solution without glucose in a sealed hypoxic GENbag fitted with a AnaeroPack (MGC, Japan) to scavenge free oxygen and the Non-OGD group was cultured in Hanks’ balanced salt solution containing the normal concentration of glucose with 5% CO_2_ for 8 h. Later, all cells were re-oxygenated and cultured in normal complete medium or were co-cultured with BMSCs. All duration were determined by our previous study [32].

### SCI model and BMSCs transplantation

Twenty-four Sprague-Dawley male rats weighing 200–220 g were divided into four groups randomly, by using the standard = RAND() function in Microsoft Excel. SCI was performed with Allen’s method in accordance with our previous study. In brief, all rats were anesthetized with pentobarbital (40 mg/kg, i.p.). Then, their vertebral columns were exposed, and laminectomy was operated at the T10 spinal vertebra. A weight of 10 g was dropped from a height of 50 mm on the exposed spinal cord. The impounder was left for 20 s to produce a moderate contusion. Immediately, the 10 μl culture medium containing 10^6^ BMSC+MOCK, BMSC+Over, or BMSC+shRNA were injected into the epicenter of the injured spinal cord using an electrode microneedle as the SCI+MOCK group, SCI+Over group, and SCI+shRNA group respectively. Meanwhile, the control rats received the sham operation with the same surgical procedure without injury, while the SCI group received the same dose of DMEM.

### Collection of BMSC-conditioned medium (BCM)

BMSCs were planted at 1 × 10^5^ cells/dish and cultured in DMEM/F12 complete medium. When attached, the cells were washed with phosphate-buffered saline (PBS) for three times and incubated in high glucose Dulbecco’s modified Eagle medium (DMEM) without serum to stimulate the production of extracellular mitochondria particles. The medium was collected 24 h later. BMSC-conditioned medium (BCM) was treated by filtering through a 1.2-μm syringe filter or by spinning cell debris down with centrifuging at 2000×*g* for 10 min. Meanwhile, BCM was filtrated through a 0.22-μm syringe filter to prepare mitochondria deleted medium (Md-BCM) that contains no extracellular mitochondria particles.

### MitoTracker red staining

BMSCs were stained with 200 nM MitoTracker Red CMXRos (Molecular Probes, M7512, Invitrogen, USA) for 30 min at 37 °C to label the intracellular mitochondria. The cells were washed three times with PBS to exclude the interference of excessive dye.

### Carboxyfluorescein succinimidyl ester (CFSE)-fluorescent label

Attached VSC4.1 motor neurons were stained by 10 μM carboxyfluorescein succinimidyl ester (CFSE, #C1031, green color, Beyotime Institute of Biotechnology, China) for 30 min in 37 °C and washed by PBS for three times.

### Co-culture of post-OGD VSC4.1 motor neurons with BMSCs

VSC4.1 motor neurons were cultured directly with BMSCs in 10-cm dishes or in the 6-well 8-μm transwell system (Corning, USA) in a 1:1 ration. All co-culture system last for 24 h.

### Microscope observation

The observation of extracellular mitochondria particles derived from BMSCs was carried out by transmission electron microscopy in accordance with previous study [33]. VSC4.1 motor neurons were cultured in 1 × 10^5^ cell/well with round coverslips and fixed with 0.5% paraformaldehyde after co-culturing. 4′,6-Diamidino-2-phenylindole (DAPI, blue color, #C1002, Beyotime Institute of Biotechnology, China) was applied to all cells to label nucleus after fixing. The internalization of extracellular mitochondria (red color) derived from BMSCs in co-cultured VSC4.1 motor neurons (green color) was captured by fluorescence microscope. To detect the regeneration of motor neuron axons, optical microscope was used to observe the length and the number of axons.

### Western blot analysis

Western blot was performed to determine the transfection efficiency of vectors and the expression level of proteins related to cellular apoptosis and mitochondrial apoptosis. Each sample was detected concentration and loaded onto 4–12% Bis-Tris gels (M00653, GeneScript, China). After the electorophoresis and transferred to PVDF membranes, the membranes were blocked in Tris-buffered saline containing 0.1% Tween 20 (TBST) and 5% skim milk (232100, BD, USA) for 90 min at room temperature. Membranes were washed with TBST and then incubated overnight at 4 °C with anti-GAPDH (1:2000, 10494-1-AP, Proteintech, USA) and anti-Bone marrow stromal cell antigen 1 (CD157) antibody (1,1000, ab208442, Abcam, USA). After being washed with TBST, membranes were incubated with infrared-labeled peroxidase-conjugated secondary antibodies for 1 h at room temperature. Bands were captured and quantificated by Odyssey CLx Image Studio (Gene Ltd., USA).

### Immunofluorescence staining

0.5% paraformaldehyde fixed cells or frozen spinal cord sections were washed 3 times in 1× PBS and blocked in blocking buffer for 60 min. Then, cells or spinal cord sections were incubated overnight at 4 °C with primary antibody as follows: rabbit anti-Grp 78 antibody (1:200, ER40402, HuaBio, CN), rabbit anti-NF-kB p65 antibody (1:200, ab16502, Abcam, USA), GAP 43 (D9C8) rabbit mAb (1:200, #8945, CST, USA), Bcl-xL (54H6) rabbit mAb (1:200, #2764, CST, USA), and AKT (phospho Thr308) antibody (1:50, om238718, Omnimabs, USA). After washing by PBS 3 times, fluorescent secondary antibodies incubated for 2 h at room temperature. Finally, DAPI was added to visualize the nucleus, and coverslips were placed

### Potential of mitochondrion measurement

The mitochondrial membrane potential (MMP) assay kit with JC-1 (C2006, Beyotime Institute of Biotechnology, China) was used to assess mitochondrial membrane potential to detect whether the extracellular mitochondrion still had function. BCM and Md-BCM were collected and mixed with JC1 (5 μM) for 30 min at 37 °C. When the membrane potential of mitochondria completely lost, green fluorescence (Ex 485 nm Em 516 nm) would be observed and the normal cells stained should show red fluorescence (Ex 579 nm/Em 599 nm). MMP was determined by the Varioskan Flash microplate reader.

### ATP measurement

Adenosine triphosphate (ATP) level was determined by CellTiter-Glo luminescence (G7570, Promega, USA). For intracellular ATP content, cells were incubated by 200 μl reagent buffer each well, standing for 30 min at room temperature for lysing cells. For extracellular ATP, the culture medium was collected and centrifuged at 2000×*g* for 10 min. Then, the supernatant was collected and centrifuged at 20,000×*g* for 20 min at 4 °C, with the remaining lower half to use. CellTiter-Glo luminescence test solution (50 μl) was added into culture media (50 μl) and incubated for 30 min at room temperature in opaque-walled 96-well plates. Luminescent signal was determined by the microplate reader.

### FACS analysis on the content of extracellular mitochondria particles

FACS analysis was performed by BD Fortessa or CytoFLEX LX. BCM were prepared and collected as described as before. Two hundred-nanometer and 300-nm diameter calibration particles, DMEM media incubating unstained BMSCs, and DMEM media were used to control for determining appropriate gates, voltages, and compensations required in multivariate flow cytometry.

### Determination of CD157/ADPR-cyclase activity

ADPR-cyclase activity was determined with nicotinamide guanine dinucleotide (NGD^+^) (N5131, Sigma, USA) as the substrate as described before [34]. Briefly, BMSCs were cultured in 96-well plate with 200 μl complete DMEM/F12 medium. After attaching, BMSCs were incubated with 500 μM NGD^+^ in 0.1 M PBS (pH 7.2) at 37 °C for 10 min, and the production was determined at excitation/emission wavelengths of Ex 290 nm/Em 410 nm with the microplate reader.

### Ca^2+^ signal detection

To explore whether the CD157/cADPR mechanism was Ca^2+^ dependent, the level of Ca^2+^ in BMSCs was determined by Fluo-4 AM (S1060, Beyotime Institute of Biotechnology, China). BMSCs were incubated in 96-well plates with 2 μM Fluo-4 AM for 30 min at room temperature. After washing with PBS for three times, fluorescence intensity was determined by the Varioskan Flash microplate reader at excitation/emission wavelengths of Ex 488 nm/Em 516 nm.

### Assessment of motor function

Basso, Beattie, and Bresnahan (BBB) locomotor scales were used to assess the recovery of rats’ motor function. The scores were obtained by two independent examiners who were blind to the four groups. All rats were observed and assessed the relevant indicators of hind limb motor function and physical control function in an open field for 3 min at 1, 7, 14, 21, and 28 days post-surgery.

### Statistical analysis

All data were expressed as mean ± SEM. Statistical analysis was analyzed by GraphPad Prism 6 software. Unpaired *t*-test was used for the analysis of two groups. Multiple comparisons were evaluated by one-way ANOVA or two-way ANOVA. *P* < 0.05 was considered to be statistically significant.

## Results

### Production and transfer of BMSC-derived mitochondria

**Fig. 1:**
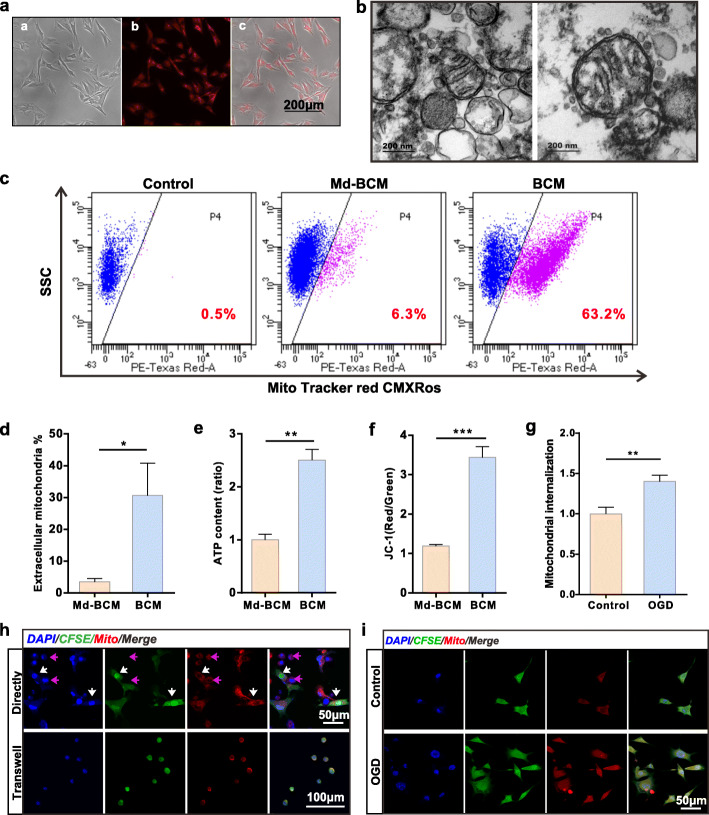
BMSCs produce functional extracellular mitochondria particles transferred into neurons. **a** Mitochondria in BMSCs visualized using Mito Tracker Red CMXRos. The red dots represent mitochondria. Scale bar, 200 μm. **b** Representative TEM images of extracellular mitochondria derived from BMSCs. Scale bar, 200 nm. **c** FACS results of extracellular mitochondria particles pre-stained using Mito Tracker Red CMXRos. Control group, BMSC medium without staining. Md-BCM group, the mitochondria depleted medium; BCM group, the normal pre-stained medium. **d** Extracellular mitochondria ratio in medium (*n* = 5). *p < 0.05. **e** Quantification of ATP content in medium (*n* = 6, fold of Md-BCM). **p < 0.01. **f** Statistics for extracellular mitochondria membrane potential (*n* = 8). ***p < 0.001. **g** Quantification of internalized mitochondria in VSC4.1 motor neurons w/o OGD (fold of control). Image J was used to measure IOD. **p < 0.01. **h** Representative fluorescence images of internalized mitochondria in VSC4.1 motor neurons co-cultured with BMSCs. In the directly group, BMSCs were pre-stained using CFSE. The purple arrows indicate VSC4.1 motor neurons and the white arrows indicate co-cultured BMSCs. Scale bar, 50 μm. In the transwell system, VSC4.1 motor neurons were pre-stained using CFSE. Scale bar, 100 μm. **i** Representative fluorescence images of VSC4.1 motor neurons. All VSC4.1 motor neurons were co-cultured with BMSCs in the transwell system. Control group, normal VSC4.1 motor neurons; OGD group, VSC4.1 motor neurons with oxygen and glucose deprivation treatment. Scale bar, 50 μm

MitoTracker Red CMXRos was employed to visualize the mitochondria of BMSCs (Fig. 1a). BMSC conditioned-medium (BCM) was collected and concentrated after 24 h of pre-staining. A transmission electron microscopy was used to observe extracellular mitochondria in BCM. The images showed that partial extracellular mitochondria maintained their normal morphology and structure (Fig. 1b). A previous study showed that the extracellular mitochondria or particle sizes ranged between 300 and 1100 nm48. Therefore, we used 0.22 μm filters to deplete BCM mitochondria to obtain the mitochondria deleted medium (Md-BCM) and then collected BCM and Md-BCM and used FACS to determine mitochondria particle content. The results showed that BCM contained a certain amount of mitochondria particles derived from BMSCs than Md-BCM (Fig. 1c, d).

We further determined the ATP content and JC-1 assay to assess the extracellular mitochondria function. Notably, when the mitochondria in extracellular particles were removed, ATP levels decreased (Fig. 1e) and MMP sharply declined when the mitochondria in extracellular particles were removed (Fig. 1f), indicating that the mitochondria are functional in the extracellular particles.

We used MitoTracker Red CMXRos to pre-stained mitochondria and CFSE to pre-stained intracellular matrix to double-stain BMSCs. BMSCs were directly co-cultured with VSC4.1 motor neurons in a 1:1 ratio for 24 h. Meanwhile, the MitoTracker Red CMXRos pre-stained BMSCs were seeded in transwell inserts and co-cultured with CFSE pre-stained VSC4.1 motor neurons in a transwell system. We then used fluorescence microscopy to confirm if the derived mitochondrion was successfully transferred into VSC4.1 motor neurons. The red spots indicated that BMSCs could produce and transfer extracellular mitochondria particles and that the mitochondria particles could be seized and initialized by VSC4.1 motor neurons (Fig. 1h). We also co-cultured BMSCs with pre-stained VSC4.1 motor neurons w/o OGD treatment and found that mitochondrial internalization in VSC4.1 motor neurons significantly increased after OGD treatment (Fig. 1g, i).

### CD157 expressions in BMSCs and VSC4.1 motor neurons

**Fig. 2:**
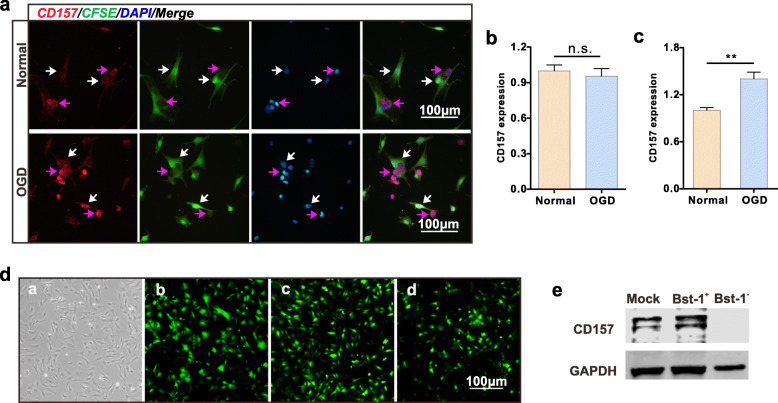
Effect of upregulated CD157 expression on BMSCs and VSC4.1 motor neurons. **a** Representative immunofluorescence images of CD157 protein. BMSCs were pre-stained using CFSE. Normal group, cells without any treatment; OGD group, VSC4.1 motor neurons were treated with oxygen and glucose deprivation for 8 h before co-cultured with BMSCs. The purple arrows indicate VSC4.1 motor Scale bars, 100 μm. **b**, **c** CD157 expression quantification (fold of normal) of VSC4.1 motor neurons (**b**) and BMSCs (**c**). Image J was used to measure IOD. n.s., non-significant, **p < 0.01. **d** Transfection effects of mock, *Bst-1*^+^, and *Bst-1*^−^ adenovirus vectors, alphabetically. All vectors were recombined with the GFP gene. Scale bar, 100 μm. **e** Western blotting of CD157 protein expression of transfected BMSCs

CD157 is expressed in both BMSCs and VSC4.1 motor neurons. We assumed that CD157 is involved in the transfer of extracellular mitochondria. However, further investigations are needed to determine whether CD157 expressions in BMSC, VSC, or both are involved in the extracellular mitochondria transfer. We used an immunofluorescence assay to determine CD157 expressions in the co-cultured BMSCs and VSC4.1 motor neurons w/o OGD. CD157 was visualized using an anti-CD157 antibody (Fig. 2a) and Image J was used to measure integrated optical density (IOD). There was no difference in CD157 expressions between OGD and normal VSC4.1 motor neurons (Fig. 2b). However, CD157 was significantly increased on BMSCs co-cultured with OGD VSC4.1 motor neurons compared with the BMSCs co-cultured with normal VSC4.1 motor neurons (Fig. 2c). These results indicated that CD157 expression in BMSCs could be involved in the regulation of BMSC-derived mitochondria production and transfer. Therefore, we constructed the bst-1+ and bst-1- adenovirus vector and observed all vectors titers using a fluorescence microscope (Fig. 2d). CD157 expression was examined using western blotting (Fig. 2e).

### Effect of CD157 on BMSC-derived extracellular mitochondria production and transfer

**Fig. 3:**
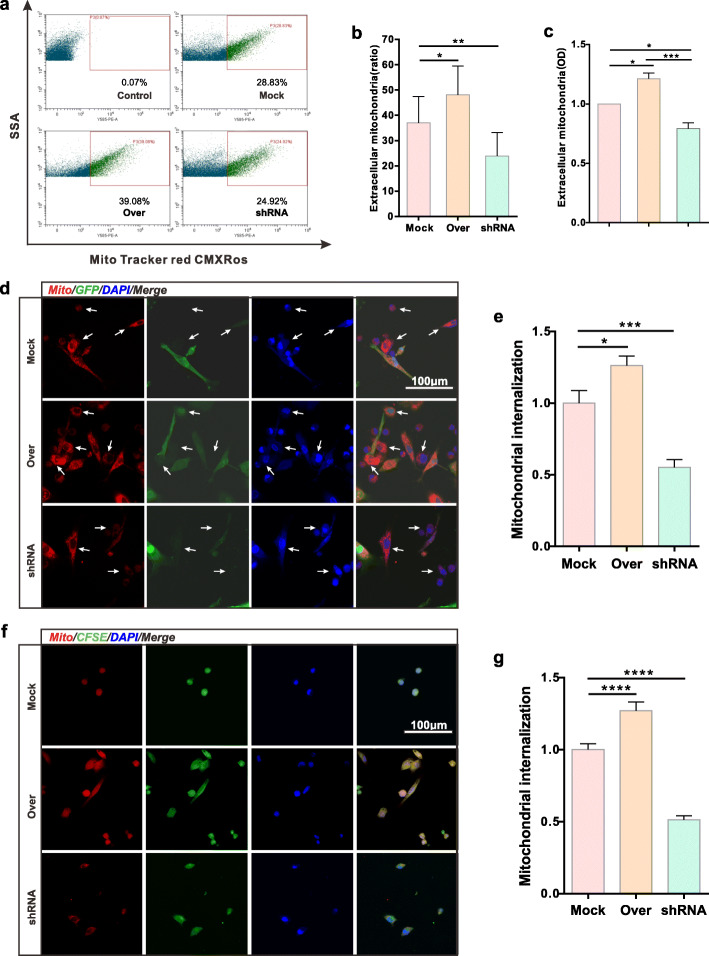
CD157 overexpression in BMSCs enhances extracellular mitochondria production and transfer. **a** FACS results of extracellular mitochondria from various transfected BMSCs. Control, BMSCs without pre-staining. **b** Extracellular mitochondria particle ratio detected by FACS (*n* = 4). *p < 0.05, **p < 0.01. **c** Extracellular mitochondria particle quantification detected by Varioskan Flash (*n* = 5). *p < 0.05, ***p < 0.001. **d** Representative confocal images of internalized mitochondria of VSC4.1 motor neurons. All VSC4.1 motor neurons were subjected to OGD treatment before direct co-culture with BMSCs. Transfected BMSCs were marked using GFP. The white arrows indicate VSC4.1 motor neurons. Scale bars, 100 μm. **f** Representative confocal images of internalized mitochondria of VSC4.1 motor neurons. OGD VSC4.1 motor neurons co-cultured with BMSCs in transwell system**.** VSC4.1 motor neurons were pre-stained using CFSE. Scale bars, 100 μm. **e**, **g** Mitochondria internalization quantification of VSC4.1 motor neurons (fold of Mock group). **c** Direct co-culture and **e** transwell co-culture. Image J was used to measure IOD. *p < 0.05, ***p < 0.001, ****p < 0.0001

We transfected BMSCs with Mock vector, overexpressed CD157 vector, and CD157 interference vectors, represented as Mock group, Over group, and shRNA group, respectively. We used pre-stained Mock, Over, and shRNA groups, as unstained BMSC groups and DMEM blank group as the control. All the BMSC conditioned-medium were collected and filtered using a 1.2-μm filter. FACS results (Fig. 3a, b) and OD value (Fig. 3c) showed that the extracellular mitochondria production was significantly upregulated in the Over group compared with the Mock group. Conversely, extracellular mitochondria production was downregulated in the shRNA group. Analysis of mitochondrial internalization via direct co-culture (Fig. 3d, e) and transwell co-culture (Fig. 3f, g) showed that mitochondria-derived from the Over group was highly internalized by injured VSC4.1 motor neurons compared with the Mock group. However, the shRNA group had a low level. Therefore, CD157 overexpression in BMSCs can upregulate BMSC-derived extracellular mitochondria particle production and transfer.

### Effects of CD157 on OGD VSC4.1 motor neurons in vitro

**Fig. 4:**
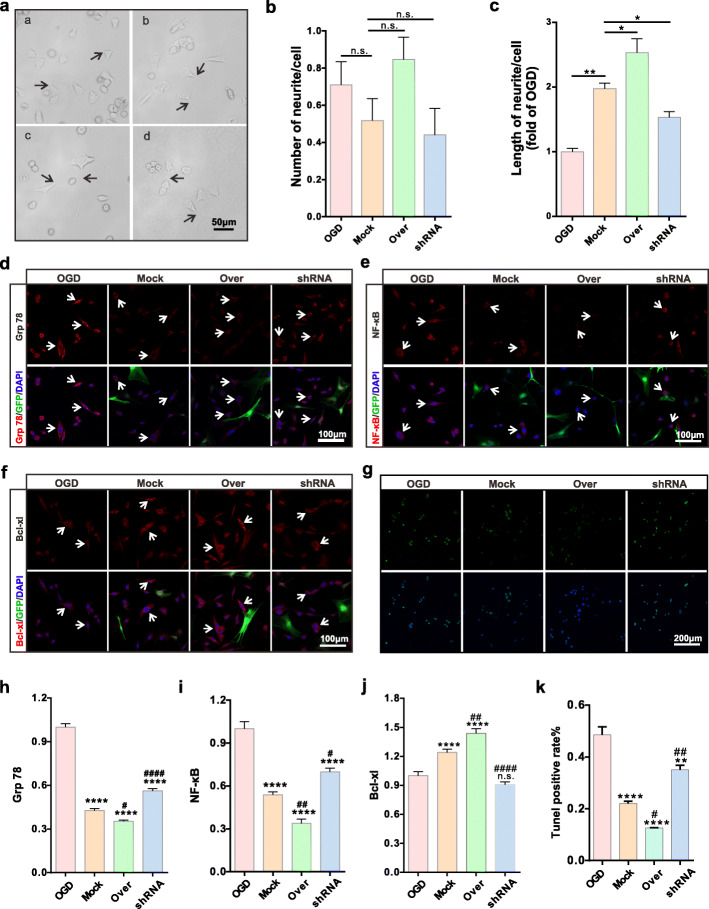
CD157 overexpression in BMSCs improves the injured VSC4.1 motor neurons in vitro. **a** Representative images of recovered VSC4.1 motor neurons. The four groups are in alphabetical order: OGD group, Mock group, Over group, and shRNA group. The black arrows show the axons. Scale bar, 50 μm. **b** Statistics for the number of neurites of each recovered VSC4.1 motor neurons. n.s., non-significant. **c** Statistics for the length of neurites of each recovered VSC4.1 motor neurons (fold of OGD group). *p < 0.05, **p < 0.01. **d**–**f** Respectively, representative immunofluorescence images of Grp 78 protein, NF-κB protein, and Bcl-xl protein of VSC4.1 motor neurons. GFP marked BMSCs. The white arrows show VSC4.1 motor neurons. Scale bars, 100 μm. **g** Representative images of TUNEL staining of VSC4.1 cells. Scale bar, 200 μm. **h**–**j** Respectively, quantification of Grp 78, NF-κB, and Bcl-xl of VSC4.1 motor neurons (fold of OGD group). n.s., non-significant, ****p < 0.0001 vs OGD group. ^#^*p* < 0.05, ^##^*p* < 0.01, ^####^*p* < 0.0001 vs Mock group. **k** Statistics of TUNEL positive rate of VSC4.1 cells. **p < 0.01, ****p < 0.0001 vs OGD group. ^#^*p* < 0.05, ^##^*p* < 0.01 vs Mock group

OGD-treated VSC4.1 motor neurons were co-cultured for 24 h with transfected BMSCs in the transwell system. We used an optical microscope (Fig. 4a) for visualization and found that CD157 expression levels in BMSCs did not affect the number of neurites (Fig. 4b). Besides, CD157 overexpression promoted the neurites’ length regeneration (Fig. 4c). Therefore, CD157 overexpression in BMSCs promotes neurite outgrowth of VSC4.1 motor neuron length and not their number.

Immunofluorescence staining was used to detect the expression of apoptosis and inflammation-related proteins, including Grp78, NF-κB, and Bcl-xl. Results showed that Grp 78 (Fig. 4d, h) and NF-κB expressions (Fig. 4f, i) were decreased in the Over group but increased in the shRNA group. However, Grp 78 and NF-κB expressions in the three groups were significantly downregulated compared to the OGD group. The expression of the anti-apoptotic protein, Bcl-xl, significantly increased in both the Mock and Over groups, compared with the OGD or shRNA groups (Fig. 4f, j). Finally, we detected the cell apoptosis via TUNEL staining and results showed that apoptosis of VSC4.1 cell in the Over group significantly decreased, compared with the other three groups (Fig. 4g, k). In summary, CD157 overexpression in BMSCs can alleviate apoptosis and inflammation of injured motor neurons.

### Effects of CD157 on spinal cord neurons in vivo

**Fig. 5:**
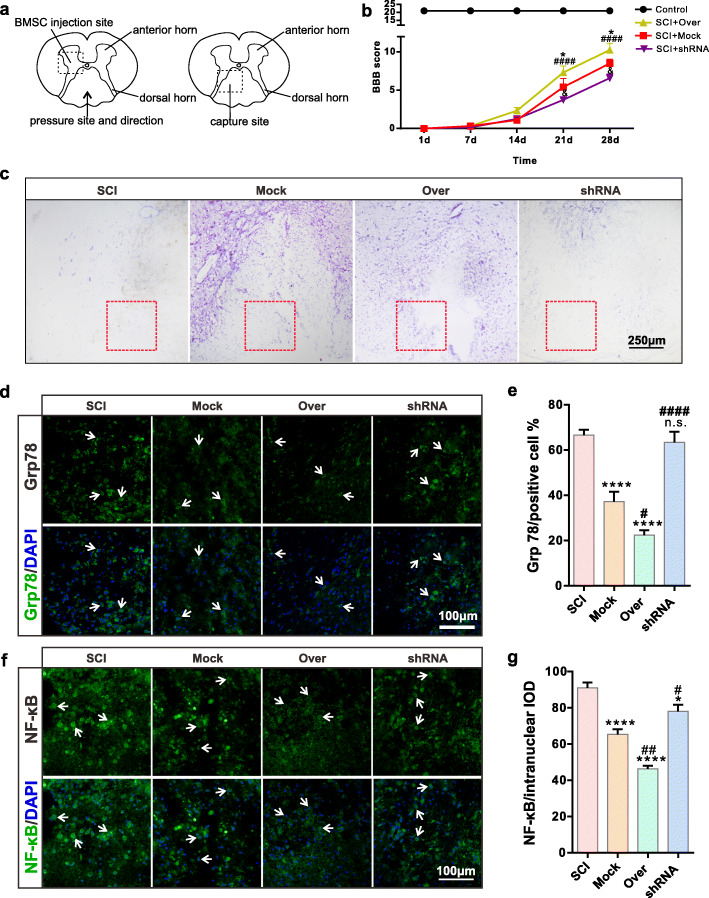
CD157 overexpression in BMSCs attenuates inflammation and apoptosis of the injured neurons in vivo (1). **a** Schematic diagram of the spinal cord. **b** BBB scores of injured rats (*n* = 6). *p < 0.05, ^&^*p* < 0.05 vs SCI+Mock group; ^####^*p* < 0.001 vs SCI+shRNA group. Representative **c** Nissl staining images of the spinal cord. The red rectangle showed the injured site. Scale bar, 250 μm. **d**, **f** Representative immunofluorescence images of Grp 78 and NF-κB of neurons at the injured site. The white arrows indicate the positive cells. Scale bars, 100 μm. **e** Statistics of Grp 78 positive rate. n.s., non-significant, ****p < 0.0001 vs SCI group. ^#^*p* < 0.05, ^####^*p* < 0.0001 vs Mock group. **g** Statistics of NF-κB intranuclear content. *p < 0.05, ****p < 0.0001 vs SCI group. ^#^*p* < 0.05, ^##^*p* < 0.01 vs Mock group

We operated in vivo BMSC-transplant surgeries after SCI on rats. The secondary injury tends to ease off at 7 days after SCI. At this time, inflammation and neuron regeneration begin to increase. Thus, we sampled and took freezing sections at 7 days after SCI (Fig. 5a). We assessed the motor function of the SCI rats. Our previous study demonstrated that normal BMSCs transplantation could facilitate rats’ motor function recovery after SCI. We divided the rats into four groups: sham operation group (control), SCI+Mock group, SCI+Over group, and SCI+shRNA group. We used BBB scores to determine the paralysis severity caused by SCI, and the scores were significantly increased in the SCI+Over group and decreased in the SCI+shRNA group (Fig. 5b).

**Fig. 6:**
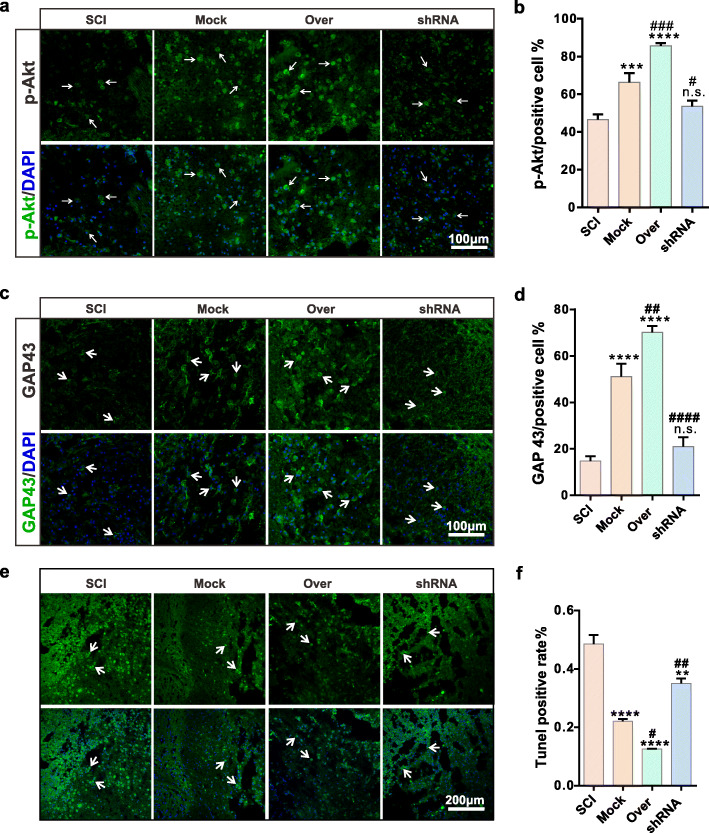
CD157 overexpression in BMSCs attenuates inflammation and apoptosis of the injured neurons in vivo (2). **a**, **c** Representative immunofluorescence images of p-Akt and GAP 43 of neurons at the injured site. The white arrows indicate the positive cells. Scale bars, 100 μm. **b**, **d** Statistics of Grp 78 and GAP 43 positive rate. n.s., non-significant, ****p < 0.0001 vs SCI group. ^#^*p* < 0.05, ^##^*p* < 0.01, ^###^*p* < 0.001, ^####^*p* < 0.0001 vs Mock group. **e** TUNEL staining images of neurons at the damage site. The white arrows indicate the positive cells. Scale bars, 200 μm. **f** Statistics of TUNEL positive rate. **p < 0.01, ****p < 0.0001 vs SCI group. ^#^*p* < 0.05, ^##^*p* < 0.01 vs Mock group

We used Nissl staining to study neuron protein synthesis and found that neurons were affected after SCI and BMSCs transplantation. There were more stained cell and deep color at the injured site in the Over group, indicating that protein synthesis enhanced in CD157 overexpressed group (Fig. 5c). We used immunofluorescence staining to detect protein expression and found Grp78 (Fig. 5d, e) and NF-κB (Fig. 5f, g) expressions were significantly decreased after overexpression transfected BMSC transplantation, while p-Akt (Fig. 6a, b) expressions increased in the Mock and Over groups. Meanwhile, the axon regeneration-related protein, GAP43, was upregulated in the Mock and Over groups, indicating that CD157 overexpression can promote neural regeneration (Fig. 6c, d). We also detected the cell apoptosis at the injured site by TUNEL staining and results showed that cell apoptosis significantly improved in Mock and Over groups (Fig. 6e, f). All above illustrated that CD157 upregulation can improve apoptosis and promote axon regeneration.

### BMSC CD157/ADPR-cyclase activity and Ca^2+^ signal

**Fig. 7:**
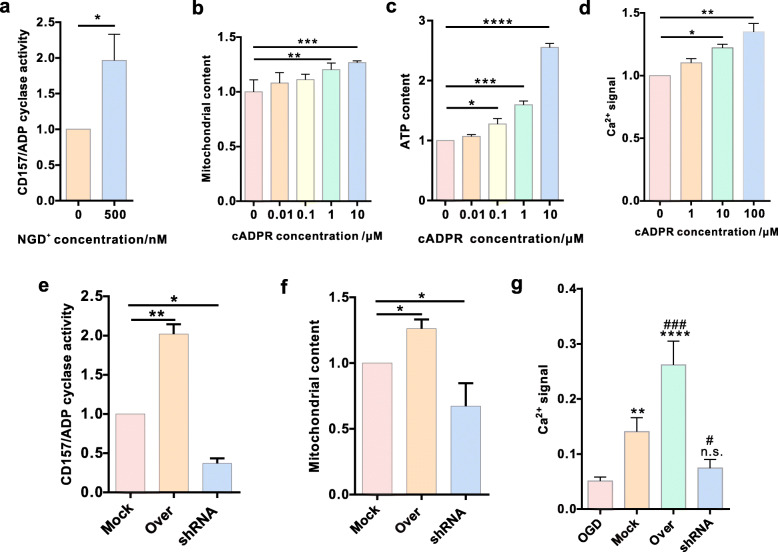
CD157/ADPR-cyclase activity and Ca^2+^ signal in BMSCs. **a** Confirmation of CD157/ADPR-cyclase activity (*n* = 5, fold of 0 nM). *p < 0.05. **b** Statistics for the mitochondria content in BMSCs pre-stained using Mito Tracker Red CMXRos (*n* = 5, fold of 0 nM). **p < 0.01, ***p < 0.001. **c** Statistics for the ATP content in BMSCs subjected to cADPR at different concentrations (*n* = 3, fold of 0 nM). *p < 0.05, ***p < 0.001, ****p < 0.0001. **d** Statistics for the Ca^2+^ signal in BMSCs subjected to cADPR at different concentrations (*n* = 3, fold of 0 nM). *p < 0.05, **p < 0.01. **e** CD157/ADPR-cyclase activity detect of different BMSCs (*n* = 3, fold of Mock group). *p < 0.05, **p < 0.01. **f** Statistics for the mitochondria content in different BMSCs (*n* = 3, fold of Mock group). *p < 0.05. **g** Statistics for the Ca^2+^ signal of transfected BMSCs when co-cultured with OGD VSC4.1 motor neurons (*n* = 4, fold of OGD group). n.s., non-significant, **p < 0.01, ****p < 0.0001 vs OGD group. ^#^*p* < 0.05, ^###^*p* < 0.001 vs Mock group

CD157 is a member of the NADase/ADP-ribosyl cyclase family. We hypothesized that the CD157-cADPR-Ca^**2+**^ signaling pathway is involved in BMSC-derived extracellular mitochondria production and transfer. We determined BMSC enzymatic activity and found that CD157 has NADase activity (Fig. 7a). We further assessed if cADPR can regulate extracellular mitochondria production. The mitochondrion measurement (Fig. 7b) and detection of ATP content (Fig. 7c) of BMSCs demonstrated that ADPR-cyclase activity regulated the extracellular mitochondria production. Then, we evaluated the cADPR-Ca^**2+**^ signaling pathway and detected calcium levels in the BMSCs and result showed that calcium concentration increased with the increasing cADPR concentration (Fig. 7d). Then, we detected the NGD^**+**^ (500 nM) treatment for various transfected BMSCs and found that BMSCs in the Over group presented highest ADP cyclase activity (Fig. 7e). Mitochondria content detect showed that BMSCs in the Over group produced more intracellular mitochondria than the other two groups (Fig. 7f). Ca^2+^ signal was also found to be highest in the Over group (Fig. 7g). Taken together, CD157-cADPR-calcium could be a potential mechanism involved in BMSC-derived extracellular mitochondria production.

## Discussion

SCI causes severe disabilities associated with motor dysfunction or paralysis and there are no effective treatment options. Secondary injury cascade, a complex, pervasive, and destructive pathological process causes a severely restricted axonal regeneration and functional recovery in SCI. Targeting a specific cascade is not an efficient therapeutic strategy because many related signaling pathways intersect and initiate other secondary injury events. Cellular energy production and metabolism take place in the mitochondrion. Mitochondrion can not only induce apoptosis but also play a key role in apoptosis [35]. Its trafficking is essential for neuronal survival, especially the axons and dendrites because of their energy demand and calcium flux [36]. Studies have reported that SCI causes mitochondria damage and disruption, by inducing oxidative stress reactions and cell apoptosis. Mitochondrial dysfunction is the initial step of neuronal injury that indirectly and directly promotes SCI pathology progression [37]. Some studies have therefore focused on the role of mitochondria in neuronal recovery and SCI treatment [38, 39]. Mitochondrial transplantation had significant benefits, including improved lower-limb locomotor function, suppressed regional endoplasmic reticulum stress, and mitochondria-dependent apoptosis inhibition [37]. Mitochondrion can therefore be a potential candidate for SCI therapy. This study observed that a portion of BMSC-derived extracellular mitochondria is important, and ATP and MMP detection confirmed that extracellular mitochondria were functional. These could be the possible mitochondrial transfer mechanism to save damaged neurons. During co-culture, the functional mitochondria were transferred to VSC4.1 motor neurons and internalized. Moreover, the injured neurons were found to internalize more exogenous mitochondria than the normal cells for rescue.

The CD157 protein of BMSCs co-cultured with OGD neurons was upregulated, indicating that CD157can generate intracellular messengers in response to stimuli, catalyzing cADPR synthesis from NAD^+^ [40]. cADPR is an important Ca^2+^-mobilizing cytosolic messenger [41] that provides the Ca^2+^ wave during fertilization [42], regulates dendritic cell functions [43], and contributes to airway disease [44]. Ca^2+^ signaling regulates some important mitochondrial activities, i.e., cytoplasmic Ca^2+^ concentration increase promotes mitochondrial ATP production [45, 46], and in turn, mitochondrial Ca^2+^ uptake regulates regulating the intracellular calcium signaling via buffering cytosolic Ca^2+^ levels [46, 47]. Research has shown that CD38 mediates extracellular mitochondria transfer in astrocytes via calcium-dependent CD38/cyclic ADP-ribose pathway, indicating that CD157 can regulate BMSC-derived extracellular mitochondria particle production and transfer.

There were elevated extracellular mitochondrial particle levels derived from the highly expressed CD157 in the modified BMSCs in separate cultures, indicating that CD157 regulates extracellular mitochondria production. Besides, the high internalization of extracellular mitochondria occurred in the CD157 overexpressed groups, after co-culturing modified BMSCs and injured neurons, showing that CD157 upregulation could be the possible cause of the high-level internalization in damaged neurons.

The in vitro experiment results showed that the regenerative synapse length of neurons were longest in the CD157 high expression group, signifying that high mitochondria internalization promotes synaptic regeneration. In this study, Grp78, NF-κB, Bcl-xl, and Akt expressions were detected both in vivo and in vitro and the results showed that the Grp78 and NF-κB expressions decreased, while Bcl-xl and p-Akt levels increased in the high CD157 group, indicating that apoptosis in the injured-cell decreases after CD157 upregulation in BMSCs. In addition, results of TUNEL detect both in vivo and in vitro directly illustrated the effect of CD157 upregulation in BMSCs on cell apoptosis. Nissl staining also indicated that the transplantation of CD157 overexpression BMSCs improved the synthesis of protein function of neurons. BBB scores also demonstrated that transplantation of the modified BMSC overexpression significantly improved the functional recovery of SCI rats.

To confirm whether the cADPR/calcium signaling pathway was involved in the process of producing and transferring mitochondria, ADPR-cyclase activity of CD157 and the mitochondria content, ATP content, and Ca^2+^ level in BMSCs treated with different concentration cADPR were measured. The cytoplasmic Ca^2+^, mitochondria, and ATP levels showed cADPR dependency. Consistently, the ADPR-cyclase activity, mitochondria content, and Ca^2+^ level presented difference in three transfected BMSCs and they were all highest in the Over group. Thus, we concluded that CD157 regulates mitochondrial production and transfer via the cADPR-Ca^2+^ pathway.

In conclusion, CD157 upregulated in co-cultured BMSCs and the high CD157 expression increases extracellular mitochondria particle production and internalization via VSC4.1 motor neurons. CD157 upregulation in BMSCs inhibits cell apoptosis and promotes the recovery of neurons and motor functions both in vivo and in vitro. However, how the SCI environment stimulates the upregulation of CD157 in BMSCs and the signaling pathways involved in this process are unknown.

## Conclusion

The present study for the first time demonstrated that CD157 on BMSCs might be stimulated by the microenvironment with injured neurons. In turn, upregulation of CD157 on BMSCs could increase the produce and transfer of mitochondria from BMSCs to injured neurons, improving the neuroregeneration and cell apoptosis. The calcium-dependent CD157/cyclic ADP-ribose signaling pathway may be involved in the mitochondria transfer from BMSCs to injured neurons of SCI rats and OGD-treated VSC4.1 motor neurons. The results suggested a novel approach for the efficiency of stem cell therapy strategies on SCI.

## Data Availability

The datasets used and/or analyzed during the current study are available from the corresponding author on reasonable request.

## Abbreviations

SCI: Spinal cord injury
BMSCs: Bone marrow mesenchymal stem cells
ATP: Adenosine triphosphate
OGD: Oxygen-glucose deprivation
MMP: Mitochondrial membrane potential
NAD: Nicotinamide adenine dinucleotide
NGD: Nicotinamide guanine dinucleotide
ADP: Adenosine–diphosphate
cADPR: Cyclic ADP-ribose
ER: Endoplasmic reticulum
BBB: Basso, Beattie and Bresnahan
BCM: BMSC-conditioned medium
Md-BCM: Mitochondria deleted BMSC-conditioned medium
FACS: Fluorescence-activated cell sorting
CFSE: Carboxyfluorescein succinimidyl ester
DAPI: 2-(4-Amidinophenyl)-6-indolecarbamidine dihydrochloride
IOD: Integrated optical density
